# Amyloid beta oligomers from human Alzheimer’s disease brain isolated by immunoaffinity chromatography

**DOI:** 10.1002/alz70861_108372

**Published:** 2025-12-23

**Authors:** Omar Orlando De Leon, Raghd Nowar, Josh Jerisha, Raquel De Campos, Kirsten L. Viola, Erika N Cline, William L. Klein

**Affiliations:** ^1^ Northwestern University, Evanston, IL USA; ^2^ Acumen Pharmaceuticals, Newton, MA USA

## Abstract

**Background:**

Over two decades of research place soluble oligomers of the amyloid beta peptide (AβOs) as pathological drivers of Alzheimer’s disease. In animal models AβOs cause memory dysfunction as well as synapse loss, tau hyperphosphorylation, neuroinflammation, and selective neuron death. How pathological activity relates to AβO structure has not been established. While it has been proposed that oligomers are without clear structure, this is not in harmony with findings that AβOs are ligands that target particular cell‐specific proteins. It is known that synthetic AβOs can comprise globular and protofibrillar forms. Which forms are targeted by therapeutic AβO antibodies in AD brain was investigated here.

**Method:**

Immunoaffinity chromatography with non‐clinical and clinical therapeutic antibodies (NU1, ACU193 and r‐mAb148, the murine precursor of Leqembi) was used to isolate AβOs; their structure was characterized by atomic force microscopy (AFM) and transmission electron microscopy (TEM).

**Result:**

As seen in synthetic preparations, human brain AβOs purified by NU1 and ACU193 chromatography were globular with diameters of 12 ± 3 nm (TEM). These were absent in non‐AD brain extracts. r‐mAb148 chromatography yielded globular and protofibrillar AβOs. R‐mAb148 but not ACU193 bound to fibrils found in synthetic preparations. A modified protocol to isolate AβOs from 5xFAD extracts using protein‐G/ACU193 columns yielded both globular and protofibrillar AβOs. The purified particles were confirmed to be AβOs by imaging antibody‐particle complexes using anti‐IgG gold, with synthetic AβOs and AβOs purified from AD brain showing the same labeling. Further validation of AβO purification was achieved in experiments showing that human brain AβOs showed specific binding to dendrites of cultured neurons.

**Conclusion:**

Immunoaffinity chromatography confirms that, as with synthetic AβOs, the AβOs in AD brain targeted by ACU193 are globular. r‐mAb148, the murine precursor of Leqembi, isolates globular and protofibrillar AβOs, extending reports that affinity purification yields fibrils from AD brain. ACU193 also recognizes protofibrils found in 5xFAD but not human brain. Experiments are in progress to evaluate the impact of chromatography method and extract preparation on particle morphology, to evaluate pathological impact in cell assays, to determine oligomer substructures by native mass spectrometry, and to determine high‐resolution structural details by cryoEM.

